# Multicentre, randomised, open-label, parallel-group, clinical phase II study to evaluate immunonutrition in improving efficacy of immunotherapy in patients with metastatic non-small cell lung cancer, undergoing systematic nutritional counseling

**DOI:** 10.1186/s12885-022-10296-x

**Published:** 2022-11-24

**Authors:** Riccardo Caccialanza, Emanuele Cereda, Francesco Agustoni, Catherine Klersy, Amanda Casirati, Elisabetta Montagna, Simona Carnio, Silvia Novello, Michele Milella, Sara Pilotto, Ilaria Trestini, Lucio Buffoni, Alessandra Ferrari, Paolo Pedrazzoli

**Affiliations:** 1 Clinical Nutrition and Dietetics Unit, Fondazione IRCCS Policlinico San Matteo, Viale Golgi 19, 27100 Pavia, Italy; 2 Medical Oncology Unit, Fondazione IRCCS Policlinico San Matteo, Pavia, Italy; 3 Biometry and Clinical Epidemiology Service, Fondazione IRCCS Policlinico San Matteo, Pavia, Italy; 4 Department of Oncology, AOU San Luigi-Orbassano, University of Turin, Turin, Italy; 5 Medical Oncology, Department of Medicine, University of Verona Hospital Trust, 37134 Verona, Italy; 6 Medical Oncology Unit, Humanitas Gradenigo, Turin, Italy; 7 Internal Medicine Department, University of Pavia, Pavia, Italy

**Keywords:** Non-small cell lung cancer, Immunonutrition, Nutritional counseling, Malnutrition, Chemotherapy, Treatment tolerance

## Abstract

**Background:**

Nutritional support, including nutritional counseling and oral nutritional supplements (ONS), has been recommended as a first-line strategy in patients with non-small cell lung cancer (NSCLC). Evidence on the efficacy of immunonutrition during immunotherapy in these patients is positive, but still limited some secondary endpoints, such as treatment toxicity and tolerance. We hypothesize that early systematic provision of ONS with a high-protein-high calorie mixture containing immunonutrients (Impact®) in addition to nutritional counseling, compared to nutritional counseling alone, is beneficial to patients with NSCLC receiving immunotherapy with or without chemotherapy.

We designed the present study to evaluate the efficacy of early systematic provision of ONS enriched with immunonutrients compared to nutritional counseling alone, in patients with NSCLC undergoing immunotherapy. Study endpoints were: treatment response (primary endpoint: progression-free survival), treatment tolerance and toxicity, body weight, body composition, protein-calorie intake, quality of life, fatigue, muscle strength and immunological profile.

**Methods:**

This is a pragmatic, multicentre, randomized (1:1), parallel-group, open label, controlled, pilot clinical trial (*N* = 180).

**Discussion:**

The improvement of efficacy of nutritional support in oncology still deserves many efforts. Immunonutrition represents a promising approach also in patients with NSCLC, but evidence on its efficacy on clinical outcomes during immunotherapy is still inconclusive. The present pilot study, which guarantees early high-quality nutritional care (assessment and treatment) to all patients in agreement with current guidelines and recommendations, could represent one of the first proofs of efficacy of early oral immunonutrition in patients with cancer undergoing immunotherapy. Further large randomized trials addressing the improvement of supportive care could be hypothesized, accordingly.

**Trial registration:**

This study is registered on ClinicalTrials.gov Identifier: NCT05384873.

## Background

Since diagnosis, patients with non-small cell lung cancer (NSCLC) frequently present a variable impairment of nutritional status. Nutritional derangements are attributable to multiple factors, including those related to tumor location as well as to systemic features, i.e. inflammatory mediators responsible for tissue wasting, anorexia and weight loss. Anticancer treatments themselves (e.g. radiotherapy, chemotherapy and surgery) can enhance the deterioration of nutritional status, by increasing energy requirements, reducing food intake and/or impairing nutrients absorption [1–3]. An altered nutritional status is associated with a worse prognosis and the more frequent need to suspend/delay anticancer therapies [4].

The international guidelines, addressing nutritional care in oncology [1–3], agree on the usefulness of nutritional support - whenever necessary - in improving clinical outcomes. Previous studies have shown that nutritional counseling is able to increase protein-calorie intake, prevent the deterioration of nutritional status and quality of life (QoL) in patients with head-and-neck (H&N) cancer [5]. Nonetheless, two recent studies suggested that, while some H&N cancer patients may present with pretreatment normal nutritional status, early nutritional counseling is essential for the improvement of treatment tolerance and survival [6, 7]. Recently, our group has demonstrated that the systematic use of oral nutritional supplements (ONS) in combination with dietary counseling in these patients enables to maintain the nutritional status, to recovery QoL and, more notably, to favor the feasibility of chemoradiotherapy (CT-RT) [8]. This effect was substantially attributed to the increase in protein-calorie intake, but the possible anti-inflammatory role of omega-3 fatty acids could not be excluded, as it is known that the modulation of inflammation by omega-3 fatty acids and other nutrients could be helpful during anticancer treatments [9].

The use of immunonutrition in patients with cancer has progressively gained attention, as a ONS enriched with immunonutrients (arginine, nucleotides and omega-3 fatty acids; Impact® - Nestlè Health Science – Creully Sur Seulles - France), was proven to be effective in reducing the risk of post-operative complications (e.g. infections, fistulas, etc.) and length of hospital stay in patients undergoing major surgery for cancer (abdominal and H&N) [10, 11]. An interest in the modulation of inflammation and immunosuppression in the tumor microenvironment is also growing [12].

Nutritional support interventions in patients with cancer should be urgently addressed through high-quality clinical trials. To date, only two trials have investigated the effectiveness of immunonutrients-enriched ONS in H&N cancer patients during adjuvant CT-RT in comparison with an isonitrogenous and isocaloric control supplement [13–15]. The immunomodulating formula failed to reduce severe mucositis during CT-RT, but a trend to improved overall survival (OS) and progression-free survival (PFS) was observed, particularly in patients more compliant to the intervention.

In recent years, along with traditional CT, immunotherapy has become the cornerstone of non-oncogene addicted advanced NSCLC treatment. However, the therapeutic efficacy of immuno-nutrient-enriched blends in patients with NSCLC undergoing immunotherapy has been not assessed yet.

The aim of the present randomized, controlled, open-label study is to assess the effect of high-protein, high-calorie ONS containing immunonutrients, in association with nutritional counseling, on immunotherapy activity and immunological profile in patients with metastatic NSCLC treated in first-line setting with immunotherapy, alone or in combination with CT.

We hypothesize that this combination will improve the efficacy of immunotherapy.

## Methods/design

The study methods and design described partially correspond to those of our ongoing trial in H&N cancer patients receiving immunonutrition [16].

### Standard protocol approval, registration, and patient consent

The study will be conducted in accordance with good clinical practice and ethical standards laid down in the 1964 Declaration of Helsinki and its later amendments.

The sudy protocol was approved by Ethics Committee of the Fondazione IRCCS Policlinico San Matteo, Pavia, Italy (12/04/2022; prot. N. 0020364/22) and was registered on ClinicalTrials.gov (NCT05384873).

A informed consent will be obtained from each patient enrolled in the study. At any time, patients will have the right to withdraw their consent without modifying their current or future care. The progresses of the study will be shared with general practitioners.

### Design

A multicentre, randomised (1:1), open-label, parallel-group, open label, controlled clinical trial will be conducted. At inclusion, patients will be allocated to study treatments using a computer-generated and centralized randomization list. The concealment will be attained through a web-based randomization.

### Subjects

#### Inclusion criteria

- Confirmed histological diagnosis of metastatic NSCLC (both squamous and non-squamous histology);
- First-line treatment with immunotherapy (alone or in combination with chemotherapy) for metastatic disease by investigators’ choice within the framework of good clinical practice and in agreement with current guidelines;
- Willingness to participate by signing written informed consent;
- Availability to administer oral supplements and immunotherapy with or without chemotherapy;
- ECOG Performance Status ≤2;
- Life expectancy ≥6 months.

### Exclusion criteria

- Age < 18 years;
- Inability to sign an informed consent;
- Indication to or ongoing artificial nutrition support (totally compromised spontaneous food-intake) and incapacity or unavailability to consume ONS.

### Assessments

Demographic and clinical data, including tumor site, histology, stage, as well as scheduled anticancer treatment, will be collected. In addition, in agreement with our ongoing trial in H&N cancer patients receiving immunonutrition [16], the following assessments will be performed:

- *Anthropometry* - according to standard procedures, body weight [to the nearest 0.1 kg] and height [to the nearest 0.5 cm] will be measured, and body mass index (BMI) will be calculated [17]. Unintentional weight loss (WL) during the last 6 month will be also recorded.
- *Calorie and protein intakes* – Intakes of energies and proteins will be estimated at all treatment visits using a 3-day quantitative food diary and the 24-hour dietary recall method (including weekdays and weekends) and by consulting validated atlas of food portions and collecting information on brand names of commercial and ready-to-eat-foods, method of preparation, and the use of dressings or added fat [18, 19]. Total protein-calorie intakes throughout the study will be estimated taking also into consideration the use of ONS. They will be considered achieved if total energy and protein consumption will attain ≥90% of estimated requirements and ≥ 1.5 g/kg/day, respectively.

Since the energy content differs between the two ONS, the difference will be taken into account in the calculation of total intakes.

- *Nutritional screening risk and malnutrition* - Nutritional risk will be evaluated at the screening visit using the Nutrition Risk Index 2002 (NRS-2002) screening tool, which is based on data collected on a routine basis (BMI, 6-month unintentional WL and oral food intake, diagnosis and age) [20]. Malnutrition will be diagnosed according to phenotypic and etiologic criteria proposed by the Global Leadership Initiative on Malnutrition (GLIM) [20].
- *Body composition* - Body composition will be assessed using bioelectrical impedance vector analysis (BIVA) and the NUTRILAB Software (Akern srl; Florence, Italy). Specifically, resistance and reactance will be measured and used to calculate phase angle (PhA), standardized PhA (SPA) and hydration index (HI) [21–24]. Operative procedures will be standardized and the same devices will be used to ensure a homogenous collection of data. The measurement of skeletal muscle mass (SMM) will be performed using computed tomography. To this purpose, muscle area will be quantified on scans at L3 and T12 [25, 26] collected at baseline disease staging and subsequent reassessments, as scheduled by the oncologists for the evaluation of response to CT. The SliceOmatic software v5.0 (TomoVision, Montreal, QC, Canada) will segment radiological images. Assessment of SMM at the level of L3 and T12 is easy, robust and a validated imaging procedure in patients with NSCLC [26].
- *Muscle strength* - A digital hand dynamometer (DynEx™, Akern / MD Systems) will be used for the measurement of muscle strength (handgrip [HG]).
- *Quality of life* - At baseline and at the end of treatment, quality of life will be evaluated using the European Organization for Research and Treatment of Cancer (EORTC) Core QoL Questionnaire (QLQ-C30), the EORTC QoL Lung Cancer 13-item module (QLQ-LC13) and the Functional Assessment of Cancer Therapy-Lung (FACT-L) Questionnaire [27, 28].
- *Fatigue* - At baseline and at the end of treatment, self-reported fatigue and its impact on activities of daily living and functional status will be assessed through the 40-item Functional Assessment of Chronic Illness Therapy – Fatigue (FACIT-F) scale [29].
- *Symptoms* - Patients will be administered the Edmonton Symptom Assessment Scale (ESAS) [30]. Presence or onset of symptoms potentially influencing food intake will be addressed accordingly.
- *Physical activity* - Self-reported physical activity level, will be assessed using the adapted version of the Godin’s Shepard Leisure Time Exercise Questionnaire before diagnosis, at baseline and post-intervention, using the adapted version of the Godin’s Shepard Leisure Time Exercise Questionnaire [31].
- *Efficacy* - Tumor evaluation will be performed during immunotherapy as per local institutional standard of care with imaging techniques; Response Evaluation Criteria in Solid Tumors (RECIST v1.1) assessments will be performed based on local institutional imaging results, using CT/MRI assessments of the brain, chest and abdomen.
- *Adverse complications and events* - All adverse complications and events attributable to nutritional interventions (namely gastrointestinal side effects), including unplanned hospitalizations and their duration, will be recorded.
- *Immunologic profile* - Measurements obtained using multiple tools will be integrated with the aim of analyzing different cell subsets, their functionality, and soluble molecules in the peripheral blood. The profile will be based on the following parameters: amount of T cells (CD3+, CD4+, CD8+), of lymphocytes, neutrophils, neutrophil to lymphocytes ratio (NLR), LDH, C Reactive Protein, T helper cells (CD4+, CD25+, FOXP3+ or CD4+, CD25hi+, CD39+), myeloid derived suppressor cells (CD11b+/CD33+/CD15+ or CD11b+/CD14+/HLADR−/CD15-), amount of plasmocytoid dendritic cells (CD303+, CD123+, CD45RA+), amount of slan+ dendritic cells (CD16 and Slan-M-DC8), IL-1β, IL-6, TNF-alfa.

A summary of study assessments and related endpoints is provided in Table 1.

**Table 1:** Assessments and related endpoints that will be investigated during the study

EVALUATIONS	T0 2 weeks before starting therapy	T1 Visit 1 Start of therapy	T2 Visit 2 Month 3	T3 Visit 3 Month 6	T4 Visit 4 Month 12 End of study
Informed consent	X				
Demographic data	X				
Inclusion/exclusion criteria	X				
Randomisation	X				
Weight history	X				
Antropometric evaluation	X	X	X	X	X
Proteic-caloric needs evaluation	X	X	X	X	X
24 hours recall	X	X	X	X	X
ESAS Scale	X	X	X	X	X
Godin’s Shepard Leisure Time Questionnaire	X		X	X	
EORTC QLQ-C30, EORTC QLQ-LC13 & FACT-L	X		X	X	X
FACIT-F	X				X
BIVA	X		X	X	X
Body composition	X		X	X	X
Immunological profile evaluation	X	X	X	X	X
Treatment efficacy			X	X	X
Adverse events		X	X	X	X
Compliance to oral nutritional supplements	X	X	X	X	X

### Anti-cancer treatments

Immunotherapy with or without chemotherapy will be prescribed by investigator’s choice within the framework of good clinical practice and in agreement with current Italian Association of Medical Oncology guidelines [32]. Particularly, first-line treatment options will consist of:

- Pembrolizumab flat dose 200 mg every 3 weeks or 400 mg every 6 weeks for patients with NSCLC harbouring PD-L1 tumor proportional score (TPS) ≥ 50%;
- Pembrolizumab flat dose 200 mg every 3 weeks + Cisplatin 75 mg/mq or Carboplatin AUC5 every 3 weeks plus Pemetrexed 500 mg/mq every 3 weeks for patients with non-squamous NSCLC harbouring PD-L1 tumor proportional score (TPS) < 50%;
- Pembrolizumab flat dose 200 mg every 3 weeks + Carboplatin AUC6 every 3 weeks plus Paclitaxel 200 mg/mq every 3 weeks or Nab-Paclitaxel 100 mg/mq on days 1, 8 and 15 every 3 weeks for patients with squamous NSCLC harbouring PD-L1 tumor proportional score (TPS) < 50%;
- Nivolumab flat dose 360 mg every 3 weeks + Ipilimumab 1 mg/kg every 6 weeks + Cisplatin 75 mg/mq or Carboplatin AUC5 every 3 weeks plus Pemetrexed 500 mg/mq every 3 weeks for patients with non-squamous NSCLC;
- Nivolumab flat dose 360 mg every 3 weeks + Ipilimumab 1 mg/kg every 6 weeks + Carboplatin AUC5 every 3 weeks plus Paclitaxel 200 mg/mq every 3 weeks for patients with squamous NSCLC.

### Nutritional interventions

Two nutritional interventions will be compared:

- Nutritional counseling + systematic provision of high-calorie, high-protein ONS containing immunonutrients (arginine, nucleotides [RNA] and omega-3 fatty acids; Impact® [237 mL per bottle]; Nestlè Health Science – Creully Sur Seulles – France; composition detailed in Table 2);
- Nutritional counseling alone.

**Table 2:** Nutrient contents of the intervention formula

Characteristic	Immunonutrition Impact®	
	1 bottle	
	100 mL	237 mL
**MACRONUTRIENTS**		
**- Proteins**, g	7.6	18
L-arginine, g	1.8	4.3
**- Carbohydrates**, g	18	44.8
**- Fats**, g	3.9	9.2
Saturated fatty acid, g	1.8	4.3
MCT/TMC, g	1.1	2.6
Mono-unsaturated fatty acids, g	0.7	1.7
Poly-unsaturated fatty acids, g	1.3	3.1
Omega-3, g	0.6	1.4
Omega-6/Omega-3 ratio, g	0.9	0.9
**- Fiber**, g	1.4	3.3
**ENERGY**		
***Total***, kcal	144	341
***% from proteins***	21	21
***% from carbohydrates***	53	53
***% from fats***	24	24
**MINERALS**		
- **Sodium**, mg	150	355
- **Potassium**, mg	190	450
- **Chloride**, mg	169	401
- **Calcium**, mg	114	270
- **Phosphorus**, mg	101	239
- **Magnesium**, mg	32	76
- **Iron**, mg	1.7	4
- **Zinc**, mg	2.1	5
- **Copper**, mcg	250	590
- **Manganese**, mcg	30	71
- **Fluoride**, mcg	21	50
- **Molybdenum**, mcg	22.5	53.3
- **Selenium**, mcg	6.6	15.6
- **Chromium**, mcg	14	33
- **Iodine**, mcg	21	50
**VITAMINS**		
- **Vitamin A**, mcg	139	329
- **Vitamin D**, mcg	0.9	2.2
- **Vitamin E (α-tocopherol)**, mg	4.2	10
- **Vitamin K**, mcg	9.4	22.3
- **Thiamine**, mg	0.17	0.4
- **Riboflavin**, mcg	25	60
- **Niacin**, mg	2.2	5.2
- **Pantothenic acid**, mcg	110	260
- **Vitamin B6**, mcg	21	50
- **Folic acid**, mcg	28	66
- **Vitamin B12**, mcg	0.8	1.9
- **Biotin**, mcg	10.1	24
- **Vitamin C**, mg	30	71
- **Choline**, mg	38	90
**- Nucleotides**, mg	180	430

Nutritional counseling is the current standard of care and will begin 2 weeks before starting immunotherapy. A registered dietitian will prepare a specific dietary program according to anthropometry, energy requirements and diet history which will be thoroughly investigated at the first visit. Dietetic plan will include both qualitative and quantitative data regarding suggested foods intake and distribution of meals. The texture of the diet will be adapted according to the presence of dysphagia for solids or liquids. Dietary prescription may include ONS, which are usually recommended when patients are unable to maintain adequate spontaneous food intake (less than 50% of the requirement for more than 1 week or only 50–75% of the requirement for more than 2 weeks) [1]. Therefore, while in the experimental arm the administration of ONS enriched with immunonutrients will begin 2 weeks before starting immunotherapy, in the control arm the use of isonitrogenous standard blend ONS will be considered only on the basis of the regular assessment of food intake. Nutritional counseling will be provided for all the length of study and will continue after the evaluation of primary endpoint according to patient’s needs. However, ONS provision will continue up to first disease re-assessment (12–14 weeks) and prolonged according to patient’s needs. Adherence to nutritional interventions will be assessed and monitored by the caregiver and the dietitian through daily recording of the bottles consumed. The safety of ONS will be also monitored by the occurrence of any potential gastrointestinal side effect.

Total daily energy requirements will be calculated by adjusting the estimated resting energy expenditure (from Harris-Benedict equation) for a correction factor of 1.5. Similarly daily protein requirements will be set at 1.5 g/kg of actual body weight. Every 7 days, a registered dietitian will perform regular consultations by face-to-face interviews and food intake will be quantified through a 3-day food diary and a 24-hour recall. The patient will have the opportunity to contact the local Clinical Nutrition Unit by telephone for any specific clarifications and advice.

During the first visit, each subject will be evaluated and consecutively allocated to one of the nutritional interventions. Data will be collected and the follow-up planned according to the checks of treatment protocol.

Stratification factors:

- Recruiting center
- Histology (squamous vs non-squamous)
- Type of treatment (immunotherapy alone vs chemo-immunotherapy)
- PD-L1 TPS (< 1% vs ≥ 1%)

It is not possible to adopt a blind design, both for the investigators and for patients. However, statistical analysis will be carried out blinded to treatment group.

### Efficacy endpoints

Primary objective: to compare immunonutrition to nutritional counseling in terms of efficacy of immunotherapy with respect to Progression-free Survival (PFS).

Primary end-point: PFS is defined as the time from randomization to the first documented progression of the disease (PD) or death due to any cause, whichever occurs first.

Secondary objectives:

- To assess the safety and tolerability of immunotherapy in association with immunonutrition compared to that observed with nutritional counseling alone;
- To assess the duration of response (DOR) with immunotherapy in association with immunonutrition compared to that observed with nutritional counseling alone;
- To compare immunonutrition to nutritional counseling in terms of changes in body composition (assessed by TC scan at L3 and T12 level and bioelectrical impedance vector analysis [BIVA]);
- To compare immunonutrition to nutritional counseling alone in terms of efficacy of immunotherapy with respect to Overall Survival (OS);
- To compare immunonutrition to nutritional counseling alone in terms of efficacy of immunotherapy with respect to quality of life (QoL);
- To compare immunonutrition to nutritional counseling alone in terms of efficacy of immunotherapy with respect to self-reported fatigue;
- To evaluate the self-reported physical activity level, before diagnosis, at baseline and post-intervention, using the adapted version of the Godin’s Shepard Leisure Time Exercise Questionnaire.

Secondary end-points:

- Adverse events (AEs) and discontinuation due to AEs;
- DOR, defined as the time from the first documented evidence of response until progression or death due to any cause, whichever occurs first;
- Changes in body composition;
- OS, defined as the time from randomization to the date of death due to any cause;
- QoL, assessed by the EORTC QLQ-C30, the QLQ-LC13 a and the FACT-L Questionnaire;
- Fatigue and its impact upon daily activities and function will be assessed at baseline and at the end of treatment using the 40-item Functional Assessment of Chronic Illness Therapy – Fatigue (FACIT-F) scale.

### Exploratory endpoints

The effects of immunonutrition on immunological profile, evaluated before, at the start and periodically during treatment (T0, 2 weeks before starting immunotherapy; T1, at baseline; T2, at 3 months; T3, at 6 months; T4, at 12 months).

The profile will be based on the following parameters: amount of T cells (CD3+, CD4+, CD8+), of lymphocytes, neutrophils, neutrophil to lymphocytes ratio (NLR), LDH, C Reactive Protein, T helper cells (CD4+, CD25+, FOXP3+ or CD4+, CD25hi+, CD39+), myeloid derived suppressor cells (CD11b+/CD33+/CD15+ or CD11b+/CD14+/HLADR−/CD15-), amount of plasmocytoid dendritic cells (CD303+, CD123+, CD45RA+), amount of slan+ dendritic cells (CD16 and Slan-M-DC8), IL-1β, IL-6, TNF-alfa.

### Benefit for participants

All participants will be receive early and tight nutritional assessment and support. Their nutritional status will be regularly monitored and nutritional support will be continuously optimized according to treatment tolerance and possible side-effects.

This study may result in significant improvements in nutritional care, which will prevent or ameliorate the impact of anticancer treatments in patients with NSCLC.

### Potential risks and burdens for research participants

No risks and burdens for participants are expected in the context of the present research.

### Dissemination

The results of the study will be presented at local, national and international medical meetings. The findings will be published in peer reviewed medical/scientific journals and made open-access on acceptance. Information may also be disseminated to the general population via public engagement and community outreach programs.

### Statistics

For a future large study, we hypothesize a difference between a proportion of success 63.5% in the control arm and of 75% in the treatment arm to be clinically relevant. This hypothesis is based on the literature (KEYNOTE-024, 042, 189 and 407 studies) [33–36].

#### Sample size

Sample size calculations are based on the primary endpoint. In a confirmatory study we would enroll 504 patients (252 in each arm), when the power is 80% and the type I error is 5%, and the proportion of success is expected 63.5% in control, and 75% in treated at 12 months.

An external pilot study of an overall trial designed with a power 80% and a type I error 5%, would aim at showing whether the treatment estimate is larger than zero. Using the one sided-90% confidence interval approach, with 154 patients (77 per arm), the lower 90% confidence limit for a zero difference would be 9.9%, excluding the 11.5% treatment effect estimate. In this case, the pilot study would point towards the presence of a treatment effect.

Accounting for a 15% dropout rate, we may enroll up to 180 patients (90 per group).

Calculations are performed following the approach by Cocks et al. [37], based on the confidence interval. Since this is a pilot study that will give elements to help in the decision to proceed with a confirmatory study, we will use a 90% one-tailed confidence interval (type I error of 10%). With this approach, the confidence interval is calculated under the H0 assumption of no difference between arms, using the expected sample size for the pilot study. If the upper limit of the interval excludes the hypothesized treatment effect in a confirmatory study, then consideration can be given to designing a confirmatory study.

#### Analysis set

##### Data analysis

The Stata software (release 17, StataCorp, College Station, TX, USA) is used for sample size calculation, generation of the randomization list and data analysis.

Being this a pilot study, all analyses are exploratory and meant to guide the decisional process to proceed to a confirmatory study. We will use the mean and standard deviation or the median and quartiles to describe continuous data and the count and percent to describe categorical data; we will report rates per 100 person year to describe time to event data. The time horizon is 12 months.

If needed, normalizing transformations will be applied to the data prior to model fitting.

##### Analysis of the primary endpoint

The risk difference between groups will be computed with a generalized liner model extended to the binomial family, with identity link (command *binreg*), together with its 90% confidence interval. Patients’ dropout is expected to be very low. For them multiple imputation of PFS will be performed using all baseline characteristics. In a second model, treatment will be adjust for Enrolling center, Histology (squamous vs non-squamous), Type of treatment (immunotherapy alone vs chemo-immunotherapy) and PD-L1 TPS (< 1% vs ≥ 1%).

#### Randomization

Patients will be randomized 1:1 by the treating physician to one of the two study arms according to a computer-generated random blocks randomization list. Randomization will be stratified by center, in order to maintain the 1:1 ratio at center level. It will be performed via web, using the REDCap at Fondazione IRCCS Policlinico San Matteo. The system will assign the patient to the treatment arm after an initial check on the eligibility criteria to be answered by the treating physician. The randomization list, with random blocks, will be generated by and is kept at the Clinical Epidemiology & Biometry Unit of the coordinating center.

The Stata software (release 16, StataCorp, College Station, TX, USA) is used for sample size calculation, generation of the randomization list and data analysis.

#### Handling of missing data and drop-outs

Patients starting artificial nutrition and patients undergoing oncologic surgery during oncological treatments qualify as dropouts.

### Study organization

The Fondazione IRCCS Policlinico San Matteo, Pavia, Italy, is responsible for the project management of the trial. The study was planned by the Clinical Nutrition and Dietetics Unit, the Medical Oncology Unit and the Clinical Epidemiology and Biometry Unit of the Fondazione IRCCS Policlinico San Matteo and the board of oncologists from other institutions listed as co-authors. Periodic board meetings will be scheduled (approximately every 3 months), in order to harmonize study procedures and to monitor and share the study progression.

### Participating institutes

Fondazione IRCCS Policlinico San Matteo, Pavia, Italy; AOU San Luigi-Orbassano, Turin, Italy; University of Verona Hospital Trust, Verona, Italy; Humanitas Gradenigo, Turin, Italy.

## Discussion

Malnutrition in oncology still represents an overlooked problem [38–40], which negatively affects clinical outcomes, and this is particularly relevant in patients with NSCLC [41]. The evidence supporting the efficacy of nutritional support in patients affected by NSCLC is promising, but still scanty and mainly focused on nutritional endpoints, while the impact on survival and treatment feasibility still requires confirmation.

Immunonutrition represents a promising approach in cancer care. It has been gaining attention in the last decades, particularly in the surgical gastrointestinal setting, where it has been demonstrated to reduce the rate of infectious complications and the length of hospitalization, without affecting mortality [42, 43].

The present study ensures the early provision of nutritional assessment and support to all the enrolled patients, in agreement with recent evidence-based guidelines and recommendations. Besides, it would help clarifying the hypothesized advantages of immunonutrition during immunotherapy for patients with NSCLC.

Toxicity frequently requires the prolongation and/or the reduction of planned systemic treatments, resulting in reduced response rates and worse prognosis [44]. Therefore, tight nutritional support with immunonutrients since treatment initiation, aimed at fully and continuously satisfying estimated energy and protein requirements, may enable not only to achieve the maintenance/improvement of nutritional status and QoL, but may also have a positive and decisive impact on the adherence to anticancer treatment and the related curative intent.

Positive results from this pilot trial would stimulate further larger randomized - hopefully international - trials, potentially resulting in the improvement of the quality of supportive care for patients with NSCLC, and in the expansion of the number of patients who may benefit from immunonutrition also in the non-surgical oncologic setting.

Finally, the immune response is emerging as a key factor affecting the efficacy of treatments also in NSCLC. Therefore, we will also evaluate how the immunological profile could change during immunotherapy according to nutritional intervention.

This approach may help to initiate the exploration of the interactions between the immune system and the supplementation with immunonutrients. This new area of research could lead to the discovery of new molecular mechanisms regulating the immune system during anticancer treatments and, potentially, the development of new therapeutic strategies aimed at enhancing the efficacy of anticancer treatments themselves.

A possible practical critical aspect of the study could be the standardization of nutritional counseling. However, to achieve this across participating center, dietitians will share their local protocols and will clarify potential discrepancies.

## Data Availability

The datasets generated and/or analyzed during the current study are not publicly available due to the Italian privacy law, but are available from the corresponding author on reasonable request.

## Abbreviations

AEs: Adverse events
BIVA: Bioelectrical impedance vector analysis
BMI: Body mass index
CT: Computed tomography
CT-RT: Chemoradiotherapy
DOR: Duration of response
ECOG: Eastern Cooperative Oncology Group Performance Status
EORTC: European Organization for Research and Treatment of Cancer
ESAS: Edmonton Symptom Assessment Scale
FACIT-F: Functional Assessment of Chronic Illness Therapy - Fatigue scale
FACT-L: Functional Assessment of Cancer Therapy-Lung questionnaire
GLIM: Global Leadership Initiative on Malnutrition
H&N: Head and neck cancer
HG: Handgrip
HI: Hydration index
L3: Third lumbar vertebra
MRI: Magnetic resonance imaging
NLR: Neutrophil to lymphocytes ratio
NRS-2002: Nutrition Risk Index 2002
NSCLC: Non-small lung cancer
ONS: Oral nutritional supplements
OS: Overall survival
PD: Progressive disease
PD-L1: Programmed Death Ligand-1
PFS: Progression-free survival
PhA: Phase angle
QLQ-LC13: Quality of life Lung Cancer 13-item module
QoL: Quality of life
RECIST: Response Evaluation Criteria in Solid Tumors
SMM: Skeletal muscle mass
SPA: Standardized phase angle
T12: Twelfth thoracic vertebra
TPS: Tumor proportional score
WL: Weight loss
